# Oxidative Stress Induces Mitochondrial Compromise in CD4 T Cells From Chronically HCV-Infected Individuals

**DOI:** 10.3389/fimmu.2021.760707

**Published:** 2021-12-08

**Authors:** Madison Schank, Juan Zhao, Ling Wang, Lam Ngoc Thao Nguyen, Dechao Cao, Xindi Dang, Sushant Khanal, Jinyu Zhang, Yi Zhang, Xiao Y. Wu, Shunbin Ning, Mohamed El Gazzar, Jonathan P. Moorman, Zhi Q. Yao

**Affiliations:** ^1^ Center of Excellence in Inflammation, Infectious Disease and Immunity, Quillen College of Medicine, East Tennessee State University, Johnson City, TN, United States; ^2^ Department of Internal Medicine, Division of Infectious, Inflammatory and Immunologic Diseases, Quillen College of Medicine, East Tennessee State University (ETSU), Johnson City, TN, United States; ^3^ Hepatitis (HCV/HBV/HIV) Program, James H. Quillen VA Medical Center, Department of Veterans Affairs, Johnson City, TN, United States

**Keywords:** HCV, oxidative stress, mitochondrial respiration, mtDNA, mtTFA

## Abstract

We have previously shown that chronic Hepatitis C virus (HCV) infection can induce DNA damage and immune dysfunctions with excessive oxidative stress in T cells. Furthermore, evidence suggests that HCV contributes to increased susceptibility to metabolic disorders. However, the underlying mechanisms by which HCV infection impairs cellular metabolism in CD4 T cells remain unclear. In this study, we evaluated mitochondrial mass and intracellular and mitochondrial reactive oxygen species (ROS) production by flow cytometry, mitochondrial DNA (mtDNA) content by real-time qPCR, cellular respiration by seahorse analyzer, and dysregulated mitochondrial-localized proteins by Liquid Chromatography-Mass Spectrometry (LC-MS) in CD4 T cells from chronic HCV-infected individuals and health subjects. Mitochondrial mass was decreased while intracellular and mitochondrial ROS were increased, expressions of master mitochondrial regulators peroxisome proliferator-activated receptor 1 alpha (PGC-1α) and mitochondrial transcription factor A (mtTFA) were down-regulated, and oxidative stress was increased while mitochondrial DNA copy numbers were reduced. Importantly, CRISPR/Cas9-mediated knockdown of mtTFA impaired cellular respiration and reduced mtDNA copy number. Furthermore, proteins responsible for mediating oxidative stress, apoptosis, and mtDNA maintenance were significantly altered in HCV-CD4 T cells. These results indicate that mitochondrial functions are compromised in HCV-CD4 T cells, likely *via* the deregulation of several mitochondrial regulatory proteins.

## Introduction

Hepatitis C virus (HCV) infection is currently an ongoing health problem worldwide. Despite highly effective direct-acting antiviral (DAA) therapies successfully improving the efficacy of HCV treatment, the annual rate of new HCV infection continues to increase, especially in individuals between the ages of 20-30, which increases the potential risk of HCV as a public health threat due to HCV-induced immune dysfunction (1–6). Individuals with chronic HCV infection often demonstrate increased oxidative stress with inflammation, disruption to mitochondrial functions, metabolic disorders, and the development of severe complications including liver cirrhosis and hepatocellular carcinoma (7–13). Furthermore, HCV proteins localize to mitochondria and contribute to increased reactive oxygen species (ROS) production and apoptosis, disrupted mitochondrial membrane potential, altered calcium homeostasis, inhibited electron transport chain (ETC) complex I, increased mitophagy, and decreased mitochondrial DNA (mtDNA) content in liver cells (10, 14). Given the role of mitochondria in regulating cellular metabolism and oxidative stress, mitochondrial dysfunction plays a pivotal role in liver-associated diseases. Beyond the detrimental effects of HCV on hepatocytes, HCV infection has also been shown to disrupt mitochondrion-dependent innate immune antiviral signaling and inflammatory pathways, leading to viral persistence (10). Several studies have detailed accelerated rates of cellular exhaustion and senescence following HCV infection in CD8 T cells, contributing to the potential reduction in viral clearance; little is known, however, about mitochondrial function following chronic HCV infection in CD4 T cells, which are essential for vaccine responses and activation of adaptive immunity (15–18). Given the high rates of blunted vaccine responses in chronically HCV-infected patients, further investigating CD4 T cells provides critical information regarding T cell dysregulation and viral persistence (2, 4–6).

We and others have previously shown primary CD4 T cell dysfunction during chronic viral infections, such as human immunodeficiency virus (HIV) and HCV, as well as accelerated rates of DNA damage, cellular senescence, apoptosis, and telomere deprotection and attrition (19–23). We have also identified a molecular link between telomeric DNA damage and mitochondrial dysfunction in primary CD4 T cells (24) as well as in a T cell line (25). Disrupted T cell homeostasis, activation status, and inflammaging could contribute to mitochondrial deregulation since mitochondria are essential to immune cell differentiation and signaling (26). Thus, CD4 T cells from chronically HCV-infected individuals may have altered mitochondrial functions that ultimately disrupt cellular metabolism, leading to CD4 T cell dysfunction. Recent evidence also implicates the role of the master regulators of mitochondrial function and biogenesis, peroxisome proliferator-activated receptor 1 alpha (PGC-1α) and mitochondrial transcription factor A (mtTFA) in cell senescence and aging (24, 27). PGC-1α and mtTFA have been shown to regulate mitochondrial biogenesis and degradation to contribute to mitochondrial homeostasis, oxidative metabolism, and mtDNA transcription (28). Furthermore, we have shown that these mitochondrial regulators are disrupted during drug- or HIV-induced telomeric injury in primary CD4 T cells (24, 29).

Because HCV infection can accelerate the aging process by inducing telomeric DNA damage, we hypothesized that the PGC-1α and mtTFA pathways may be dysregulated during chronic HCV infection and thus may contribute to mitochondrial compromise. In this study, we examined mitochondrial fitness and regulators in primary CD4 T cells from HCV patients. We provide evidence that mitochondrial functions are disrupted in CD4 T cells during chronic HCV infection. We found that silencing mtTFA reduced cellular respiration and mtDNA content. These results suggest that CD4 T cell mitochondrial function and cellular metabolism are compromised during chronic HCV infection, which contribute to aberrant T cell homeostasis and immune failure.

## Materials and Methods

### Study Subjects

The study subjects included two populations: 42 chronically HCV-infected individuals and 44 age-matched healthy subjects [HS; negative for HBV, HCV, and HIV infections, obtained from BioIVT (Gray, TN)]. All HCV patients were positive for HCV RNA and were collected prior to DAA treatment. All patients provided written informed consent. All experiments were conducted according to the procedures approved by the ethics committee of the joint Institutional Review Board (IRB) of East Tennessee State University and James H. Quillen VA Medical Center (ETSU/VAMC). Subject characteristics are shown in **Table 1**.

**Table 1:** Characteristics of the study subjects.

Subjects	*n*	Gender	Mean Age	Median Viral Load
HS	52	37M/15F	44 (22-66)	N/A
HCV	49	40M/9F	49 (27-71)	2,550,389 (3,020-12,000,000)

### Cell Isolation and Culture

Peripheral blood mononuclear cells **(**PBMCs) isolation and CD4 T cell purification were performed as described previously (29). CD4 T cells were cultured in RPMI-1640 medium containing 10% fetal bovine serum (FBS) (Atlanta Biologicals; Flowery Branch, GA), 100 IU/ml penicillin, and 2 mM L-glutamine (Thermo Fisher; Waltham, MA). For T cell receptor (TCR) stimulation, CD4 T cells were cultured in the presence of 2 μg/ml anti-CD3 and 4 μg/ml anti-CD28 for 3 days in the presence of 30 IU/ml IL-2.

### Flow Cytometry

For T cell phenotype analysis, the following fluorescence-conjugated antibodies were used: CD4-PE (Cat# 300508) or CD4-FITC (Cat# 300506) with CD45RA-PerCP (Cat# 304156) and CD71-A647 (Cat# 334118, BioLegend; San Diego, CA). For intracellular staining, the cells were fixed and permeabilized with the Foxp3 Transcription Factor Staining Buffer Set (Cat# 00-5523-00) (Invitrogen; Carlsbad, CA), followed by staining for PGC-1α-A488 (Cat# NBP1-04676AF488, Novus Biologicals; Littleton, CO) or mtTFA-A488 (Cat# ab198308, Abcam; Cambridge, MA) for 45 min at room temperature.

For mitochondrial function analysis, MitoTracker Orange (MO) (Cat# M-7511) and MitoTracker Green (MG) (Cat# M-7514, Invitrogen) probes were used according to the manufacturer’s protocol. 100 nM MG and 500 nM MO were cultured with purified CD4 T cells for 30 min at 37°C and then analyzed by flow cytometry. Mitochondrial ROS was assessed following 3 day TCR stimulation, by staining with 5μM MitoSOX Red mitochondrial superoxide indicator (Cat# M36008, Invitrogen) at 37°C for 30 min, and washing with DPBS 3 times. Controls for these assays included unstained, isotype controls, and single positive staining, which were used for gating and compensation. Samples were analyzed by a BD AccuriC6 Plus flow cytometer and FlowJo V10 software.

### mtDNA Copy Number

Genomic DNA was extracted from TCR-stimulated CD4 T cells using the pureLink genomic DNA kit (Thermo Fisher). PCR primers included mtDNA tRNALeu Forward: 5′-GGTCCCCAATCACCTCATCTG-3′; mtDNA tRNALeu Reverse: 5′-TGGCCATGGGTATGTTGTTA-3′; nuDNA β2-microglobulin 5′- TGCTGTCTCCATGTTTGATGTATCT-3′; and nuDNA β2-microglobulin Reverse: 5′- TCTCTGCTCCCCACCTCTAAGT-3′. The quantification of mtDNA copy number was performed as described previously (24, 30).

### mtTFA Knockdown

CD4 T cells were purified from PBMCs isolated from HS and TCR stimulated for 3 days. mtTFA crRNP was generated following a previously published protocol (31) and used for transfection using the P3 Primary Cell 4D nucleofector X Kit L (Cat# V4XP-3024, Lonza; Basel, Switzerland) and program EH115. The cells were harvested at day 3 for analysis.

### Western Blotting

Western blot analysis was performed as described previously (24). Primary antibodies included mtTFA (Cat# 8076, Cell Signaling; Danvers, MA). Appropriate horseradish peroxide-conjugated secondary antibodies (Cell Signaling) were used. The protein bands were captured and analyzed by the Chemi Doc Imaging System (Bio-Rad) and normalized to β-Actin (Cat# 12262).

### Seahorse XFp Cell MitoStress Tests

Seahorse XFp Cell Mito Stress Tests (Agilent Technologies; Santa Clara, CA) were completed using an XFp instrument and the Cell Mito Stress Test kit following the manufacturer’s instructions as described previously (24). Data were analyzed using the Seahorse Wave software.

### Liquid Chromatography-Tandem Mass Spectrometry (LC-MS/MS)

Mitochondria were fractionated from CD4 T cells isolated from 3 HS and 3 HCV subjects using the Qproteome mitochondria isolation kit (QIAGEN; Germantown, MD). Approximately 5 μg mitochondrial proteins were assessed using an LC-MS shotgun approach at the Center for Integrative Proteomics Research of the Robert Wood Johnson Medical School and Rutgers University following their standard protocol (32). Label-free quantification (LFQ) was used to determine the relative protein amounts as a fold change.

### Statistical Analysis

The data were analyzed using Prism 7 software and are presented as mean ± SEM. The outliers were identified by the ROUT method (Q = 1.000%) and excluded from the analysis. Comparisons between two groups for normal distribution were made using a parametric paired or unpaired t-test (equal SDs) or parametric unpaired t-test with Welch’s correction (unequal SDs). Comparisons between two groups for skewed distribution data were made using the nonparametric Wilcoxon paired t-test or nonpaired Mann-Whitney U test. *P*-values of <0.05 were considered statistically significant.

## Results

### Intracellular ROS and Mitochondrial Mass Are Dysregulated in CD4 T Cell Subpopulations From HCV Patients

The most commonly described mitochondrial abnormalities during chronic HCV infection include increased mitochondrial ROS production, inhibition of mitochondrial electron transport, alterations in mitochondrial morphology, inhibition of innate immune responses, and sensitization to mitochondrial cell death cascades (10, 11, 33, 34). We have previously shown increased oxidative stress, apoptosis, and DNA damage, as well as shortened telomeres in CD4 T cells from chronically HCV-infected individuals compared to age-matched HS (19, 20, 22, 23). We and others have also demonstrated dysregulated T cell homeostasis of CD4 T cell subpopulations, including naïve (CD45RA^+^), memory (CD45RA^-^), cycling (CD71^+^), and non-cycling (CD71^-^) cells following chronic viral infection (19, 20, 29, 35). Given the critical role of mitochondria on cellular metabolism and viability, we examined T cell homeostasis and mitochondrial functions in CD4 T cell subpopulations in PBMCs by measuring MO for the level of intracellular ROS and MG for mitochondrial mass.

**Figure 1:**
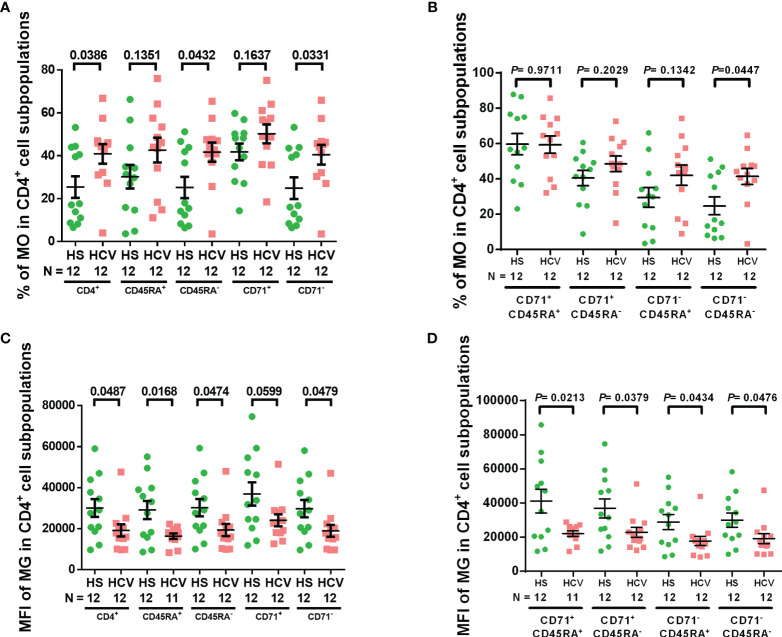
CD4 T cell intracellular ROS and mitochondrial mass in HCV-infected individuals. **(A, B)** Frequency of MitoTracker Orange^+^ (MO^+^) cells in total CD4^+^, CD4^+^ CD45RA^+^, CD4^+^ CD45RA^-^, CD4^+^ CD71^+^, CD4^+^ CD71^-^, CD4^+^ CD71^+^ CD45RA^+^, CD4^+^ CD71^+^ CD45RA^-^, CD4^+^ CD71^-^ CD45RA^+^, CD4^+^ CD71^-^CD45RA^-^ cell subpopulations from HCV-infected individuals and health subjects (HS). **(C, D)** MFI of MitoTracker Green (MG) in total CD4^+^, CD4^+^ CD45RA^+^, CD4^+^ CD45RA^-^, CD4^+^ CD71^+^, CD4^+^ CD71^-^, CD4^+^ CD71^+^ CD45RA^+^, CD4^+^ CD71^+^ CD45RA^-^, CD4^+^ CD71^-^ CD45RA^+^, CD4^+^ CD71^-^ CD45RA^-^ cell subpopulations from HCV-infected individuals and HS. The data were analyzed by parametric unpaired T-tests with or without Welch’s corrections or nonparametric Mann-Whitney U tests.

We have previously shown increased ROS in naïve CD4 T cells from chronically HCV-infected individuals (19). Here we first assessed the intracellular ROS levels by staining with MO, which is a marker of intracellular ROS (36, 37) as well as mitochondrial membrane potential, and is retained after the loss of membrane integrity (38), in CD4 T cell subpopulations from chronically HCV-infected individuals. **Supplemental Figure 1** shows representative dot plots for the gating strategy to determine the frequency of MO^+^ cells in each subpopulation. Singlets were gated, followed by scatter, CD4^+^ cells, CD45RA^+/−^ naïve/memory cells, CD71^+/−^ cycling/non-cycling cells, and then MO^+^ cells in these populations. We observed significant increases in MO frequency in total, memory (CD45RA^-^), non-cycling (CD71^-^), and non-cycling memory (CD71^-^ CD45RA^-^) CD4 T cell subpopulations from chronically HCV-infected patients versus HS (**Figures 1A, B**). These results indicate elevated ROS production and compromised mitochondrial membrane potential in CD4 T cell subpopulations during HCV infection.

MG is a selective dye that binds to free thiol groups of cysteine residues, which are naturally enriched in mitochondrial proteins, and thus is a measurement of mitochondrial density/mass. As shown in **Figures 1C, D**, the MFI of MG was significantly decreased in total, naïve, memory, non-cycling, cycling naïve, cycling memory, non-cycling naïve, and non-cycling memory CD4 cell subpopulations from HCV patients - similar to our previous findings in CD4 T cells from ART-controlled people living with HIV (PLHIV) (29). These results suggest that mitochondrial biogenesis and fitness are reduced in CD4 T cells from chronically HCV-infected individuals.

### Mitochondria From HCV Patients Have Increased ROS and Decreased mtDNA Content

**Figure 2:**
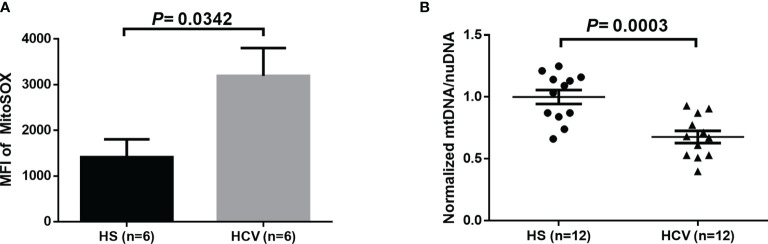
Mitochondrial-specific ROS and mitochondrial DNA (mtDNA) copy number in TCR-stimulated CD4 T cells from HCV-infected individuals and HS. **(A)** Flow cytometry analysis of the MFI of MitoSOX in TCR-stimulated CD4 T cells from HCV-infected individuals and health subjects (HS). **(B)** Quantification of mitochondrial DNA (mtDNA)/nuclear DNA (nuDNA) content in TCR-stimulated CD4 T cells from HCV-infected individuals, normalized to HS. The data were analyzed by parametric unpaired T-tests.

Given the evidence of HCV induction of ROS in liver cells and increases of MO in CD4 T cells from HCV patients (11, 18, 19, 39), we determined whether HCV infection can induce increased mitochondrial-specific ROS in CD4 T cells from HCV patients. As shown in **Figure 2A**, we observed a significant increase in the MFI of MitoSOX in CD4 T cells from HCV patients following 3-day TCR-stimulation. Since mitochondrial ROS can induce mtDNA damage, we also evaluated if mtDNA copy number is affected during HCV infection. **Figure 2B** shows significantly reduced mtDNA content in TCR-stimulated CD4 T cells from HCV patients, as determined by real-time qPCR.

Due to the critical role of mitochondria in regulating cellular metabolism and respiration, we next used Seahorse Cell MitoStress tests to evaluate the oxygen consumption rate (OCR) in CD4 T cells from HCV patients versus HS. However, we did not observe any significant changes in the OCR in either unstimulated (**Supplemental Figure 2A**) or TCR-stimulated (**Supplemental Figure 2B**) CD4 T cells from HCV patients versus HS, indicating that mitochondrial respiration is not significantly impacted by the excessive oxidative stress in the setting of chronic HCV infection. While dysregulated cellular respiration can provide a functional readout for mitochondrial metabolic and energetic output, it is possible that mitochondrial respiratory functions are compensated during chronic HCV infection, or that the alterations in mitochondrial-specific ROS and reduced mtDNA levels may be sufficient to induce mitochondrial dysfunction.

### Expressions of PGC-1α and mtTFA Are Remarkably Suppressed in CD4 T Cell Subpopulations From HCV-Infected Individuals

**Figure 3:**
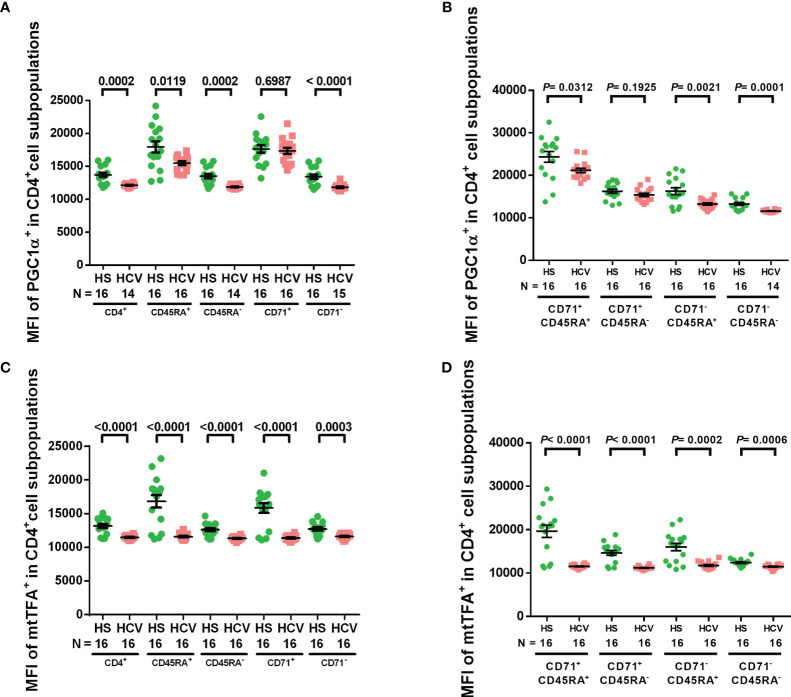
PGC-1α and mtTFA expressions in CD4 T cells from HCV-infected individuals and HS. **(A, B)** Flow cytometry analysis of the MFI of PGC-1α^+^ cells in total CD4^+^, CD4^+^ CD45RA^+^, CD4^+^ CD45RA^-^, CD4^+^ CD71^+^, CD4^+^ CD71^-^, CD4^+^ CD71^+^ CD45RA^+^, CD4^+^ CD71^+^ CD45RA^-^, CD4^+^ CD71^-^ CD45RA^+^, CD4^+^ CD71^-^ CD45RA^-^ cell subpopulations from HCV-infected individuals and health subjects (HS). **(C, D)** Flow cytometry analysis of the MFI of mtTFA^+^ cells in total CD4^+^, CD4^+^ CD45RA^+^, CD4^+^ CD45RA^-^, CD4^+^ CD71^+^, CD4^+^ CD71^-^, CD4^+^ CD71^+^ CD45RA^+^, CD4^+^ CD71^+^ CD45RA^-^, CD4^+^ CD71^-^ CD45RA^+^, CD4^+^ CD71^-^ CD45RA^-^ cell subpopulations from HCV-infected individuals and HS. The data were analyzed by parametric unpaired T-tests with or without Welch’s corrections or nonparametric Mann-Whitney U tests.

We have previously shown downregulation of the master regulators of mitochondrial function and metabolism, PGC-1α and mtTFA, in CD4 T cells treated with a telomere-targeting drug (sodium meta-arsenite (KML001)) and CD4 T cells from ART-controlled PLHIV (24, 29). To determine whether chronic HCV infection impacts these mitochondrial regulators, we examined PGC-1α and mtTFA expressions in CD4 T cell subpopulations from HCV patients and HS by flow cytometry. We found that the MFI of PGC-1α^+^ cells was significantly reduced in total, naïve, memory, non-cycling, cycling naïve, non-cycling naïve, and non-cycling memory CD4 T cell subpopulations from HCV-infected individuals (**Figures 3A, B**), while the MFI of mtTFA^+^ cells was significantly reduced in all CD4 T cell subpopulations from HCV patients versus HS (**Figures 3C, D**). These findings suggest that chronic HCV infection significantly suppresses the expressions of master regulators of mitochondrial activity, PGC-1α and mtTFA, which could underlie HCV-induced mitochondrial compromise.

### mtTFA Regulates Mitochondrial Functions *via* mtDNA Maintenance in CD4 T Cells

**Figure 4:**
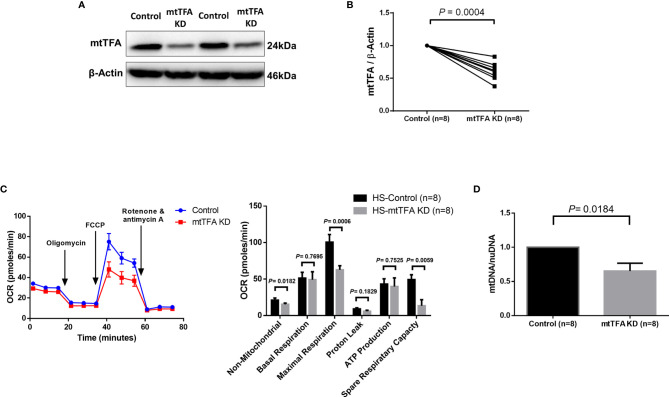
mtTFA Knockdown in CD4 T cells from HS. **(A, B)** Representative Western blots and summary data of mtTFA and β-actin expression in HS CD4 T cells following CRISPR/Cas9 TFAM KD. **(C)** Representative OCR summary data for non-mitochondrial, basal respiration, maximal respiration, spare respiration capacity, proton leak, and ATP production in CD4 T cells with or without TFAM KD. **(D)** Quantification mitochondrial DNA (mtDNA)/nuclear DNA (nuDNA) content in HS CD4 T cells with or without TFAM KD. DNA content from mtTFA KD was normalized to the control group. KD, knockdown. The data were analyzed by parametric paired T-tests.

Since mtTFA regulates mtDNA replication and maintenance, we sought to investigate the role of mtTFA in regulating mitochondrial functions *via* mtTFA knockdown in CD4 T cells from HS using the CRISPR/Cas9 technique (31). We observed significantly reduced mtTFA protein levels following the knockdown (**Figures 4A, B**). The mtTFA knockdown led to a significant impairment of the cellular non-mitochondrial and maximal respiration, as well as spare respiratory capacity (**Figure 4C**). mtTFA knockdown also led to a significant reduction in mtDNA copy number relative to nuclear DNA (nuDNA) content (**Figure 4D**). Collectively, these results implicate a critical role of mtTFA in maintaining normal cellular respiration and mtDNA replication in healthy CD4 T cells, and thus, its inhibition may lead to abnormal mitochondrial functions during HCV infection.

### Expressions of Mitochondrial Antioxidant, mtDNA Maintenance, and Cell Death Proteins Are Disrupted in HCV-CD4 T Cells

**Figure 5:**
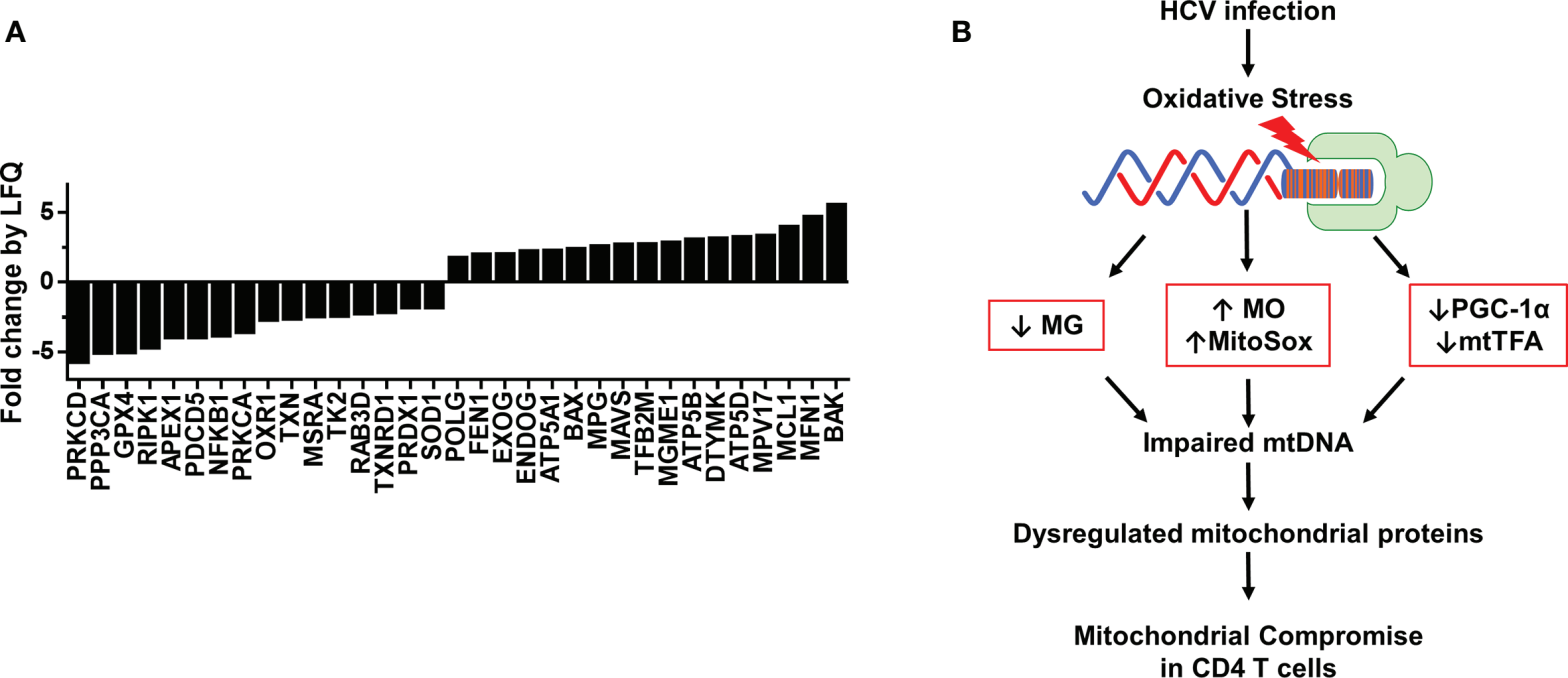
Proteomic analysis of mitochondria from CD4 T cells of HCV-infected individuals and HS. **(A)** Fold change by quantification (LFQ) of proteins isolated from mitochondria of HS (n=3) and chronic HCV patients (n=3) was determined by LC-MS proteomic analysis. **(B)** A model of HCV infection-induced oxidative stress that contributes to the alterations in MG, MO, MitoSOX, PGC-1α and mtTFA expressions, which further impair mtDNA and contribute to dysregulated mitochondrial proteins, ultimately leading to compromised mitochondrial function in CD4 T cells during chronic viral infection.

Based on our finding that HCV infection dysregulates master regulators of mitochondrial function, we were intrigued to identify other mitochondrial proteins that may be impacted during chronic HCV infection. Thus, we investigated the protein composition of mitochondria isolated from CD4 T cells of 3 HCV-infected patients and 3 HS using LC-MS. We screened 1,023 known mitochondrial localized proteins and observed > 2-fold change in 466 proteins between HCV and HS. Based on protein function descriptions in the Human Gene Database and Uniport, we selected those proteins with known roles in regulating oxidative stress, DNA damage repair, mtDNA content, cell death, and cell activation. This analysis identified 33 mitochondrial localized proteins of interest that are altered during HCV infection (**Figure 5A**).

The proteomic analysis revealed repression of proteins critical to ATP synthesis, including ATP5A1, ATP5B, and ATP5D, as well as several critical antioxidant proteins, including GPX4, OXR1, TXN, MSRA, PRDX1, and SOD1. These changes could contribute to the drastic increases in oxidative stress we observed in CD4 T cells during HCV infection. We also found disruptions in DNA damage repair pathway proteins, including APEX1, POLG, FEN1, and MPG, as well as proteins specific for mitochondrial DNA synthesis and repair, such as TK2, RAB3D, EXOG, ENDOG, TFB2M, MGME1, DTYMK, and MPV17. Moreover, the expressions of mitochondrial proteins associated with regulating cell death, including RIPK1, PDCD5, PRKCA, BAX, MCL1, MFN1, and BAK were also altered by chronic HCV infection. Furthermore, the mitochondrial expressions of proteins involved in cellular signaling and facilitating immune cell activation and antiviral immunity, such as PRKCD, PPP3CA, NFKB1, TXNRD1, and MAVS, were disrupted in CD4 T cells from chronically HCV-infected individuals compared to HS. Based on these changes in the levels of mitochondrial antioxidant proteins and the dysregulated expression of proteins involved in the base excision repair (BER) pathway and mtDNA regulation, our studies suggest that HCV deregulates the expression of antioxidant proteins, allowing oxidative stress to accumulate and induce additional genomic and mtDNA damages, leading to mitochondrial compromise. Collectively, these results suggest that HCV infection deregulates the expression of multiple mitochondria-localized proteins, which contributes to mitochondrial compromise in CD4 T cells during viral infection.

## Discussion

Aberrant T cell function is a critical factor in the progression and pathogenesis of a wide range of viral diseases. Despite well-controlled infection by DAA therapy, chronically HCV-infected individuals show over-activation and increased DNA damage in immune cells, as well as an increased risk of developing chronic liver disease and hepatocellular carcinoma (7–12, 19). While research shows increased incidences of metabolic disorders in chronically HCV-infected individuals, the underlying mechanisms leading to perturbations of cellular metabolism remain poorly understood. In this study, we investigated HCV-induced mitochondrial compromise under oxidative stress in primary CD4 T cells during chronic viral infection. Our results demonstrate that CD4 T cells have compromised mitochondria following chronic HCV infection, as evidenced by the upregulation of intracellular and mitochondrial ROS (MO and MitoSOX), downregulation of mitochondrial mass (MG) and mtDNA copy number, and dysregulation of expression of the master regulators of mitochondrial function/biogenesis PGC-1α and mtTFA. We showed that knockdown of mtTFA impairs mitochondrial respiration and reduces mtDNA copy number. We also observed disrupted expression of mitochondrial proteins involved in regulating cellular metabolism and defense. Based on these results, we propose a model (**Figure 5B**) where HCV infection induces increased intracellular ROS, which in turn disrupts cellular signaling, mtDNA maintenance, and mitochondrial proteins, leading to mitochondrial compromise and ultimately impaired CD4 T cell function.

Mitochondrial deregulation induced by HCV infection leads to excessive oxidative stress, which contributes to systemic inflammation, altered immune cell function, and viral persistence (10, 14). Immune cells naturally undergo metabolism fluctuations to combat their continually changing energetic demands. Specifically, immune cells undergo a significant shift in their metabolic profile to facilitate immune cell activation, differentiation, and survival. For instance, T cell metabolism differs between resting and activated cells, where resting cells primarily utilize OXPHOS and activated cells shift to a glycolytic mechanism to maintain energetic needs under activation/stimulation (9). Research highlights the role of mitochondria in regulating macrophage polarization and the release of pro-inflammatory cytokines (40). In line with this, we have previously shown that HCV infection can induce CD4 T cell dysfunction, characterized by aberrant cell differentiation, ROS overproduction, accelerated DNA damage and telomere attrition, increased cellular senescence, impaired DNA damage repair pathways, and increased apoptosis (19, 22, 23, 41).

Recently, we have shown CD4 T cell hyperactivation of Akt/mTOR/pS6 signaling following TCR stimulation, which in turn led to elevated expression of inflammatory cytokines and increased DNA damage and cellular apoptosis (41). Other studies revealed that inhibition of mTOR signaling can reduce ROS production and restore T cell functions (42, 43). ROS are by-products of normal cellular metabolism and participate in cellular signaling pathways related to cellular apoptosis, cytokine, cell growth factor, and hormone release (44). Intracellular ROS are critical to natural T cell activation and proliferation (45, 46). Furthermore, recent studies support the notion that mitochondrial ROS production also plays a major role in inflammatory cytokine production and immune cell activation (40, 47). Mounting evidence supports that HCV can induce oxidative stress by a variety of mechanisms (34, 44). For example, HCV core protein has been shown to regulate the release of mitochondrial ROS *via* an increase in calcium uptake, which disturbs the ETC and electron leakage (8, 34, 48). Moreover, HCV has been shown to disrupt antioxidant enzyme activity, leading to excessive oxidative stress (44, 49–51). Evidence suggests that HCV alters normal cellular signaling leading to increased DNA damage, cell cycle arrest, apoptosis, inflammasome activation, etc. (33, 52–55). More investigations are needed to further evaluate the underlying mechanisms by which ROS contributes to mitochondrial compromise and influences immune cell function in CD4 T cells.

Chronic HCV infection induces genomic and mtDNA damage and also disrupts DNA damage repair pathways *via* oxidative stress (19, 20, 22, 23). Additionally, evidence shows deregulated expression of ataxia-telangiectasia mutated (ATM), telomeric repeat binding factor 2 (TRF2), and topoisomerase I/IIA (Top1/2α), along with mitochondrial compromise, in T cells from chronically HCV-infected individuals (19, 22, 23, 56). We have previously identified a cellular mechanism by which a telomere-targeting drug (KML001) specifically induces telomeric DNA damage and attrition, as well as mitochondrial compromise mediated by p53 suppression of the PGC-1α pathway in primary CD4 T cells (24, 57). Furthermore, we and others have shown a crosstalk mechanism where direct targeting of either telomeres or mitochondria *via* a chemoptogenic tool that selectively produces a single oxygen (^1^O_2_) at their distinct locations disrupts mitochondrial functions or induces telomeric DNA damage and shortening, respectively (25, 58, 59). HCV-CD4 T cells have significantly shortened telomeres with DNA damage due to the inhibition of the telomere shelterin protein TRF2 (19). Interestingly, CD4 T cell telomere length correlates with fibrosis level, treatment response, hepatic cell degradation, and ultimately clinical outcome (60). Collectively, these studies present the possibility that both telomeres and mitochondria become disrupted, leading to impaired T cell metabolism and immune function during chronic HCV-infection. We have previously shown that mtTFA is repressed in CD4 T cells from virally suppressed PLHIV, similar to that observed in this study during HCV infection (29). Specifically, mtTFA is critical for facilitating mtDNA maintenance. Given the role of mtTFA in regulating mitochondrial functions and biogenesis, the disruption of mtTFA protein expression likely contributes to the impairment of mtDNA. Upon accumulation of a significant level of mtDNA damage, the cell respiratory chain can become disrupted, resulting in significant mitochondrial failure (61, 62). This may explain the significant impairment in mitochondrial oxygen respiration following mtTFA knockdown in CD4 T cells from HS, which exemplifies a direct model of impaired mtDNA replication. However, the impairment in mitochondrial respiration was not observed in CD4 T cells from chronic HCV patients, which represent a chronic condition where mitochondrial function may show partial compensation over a prolonged period.

Several variables may contribute to mitochondrial dysfunction during HCV infection. For instance, DAA therapies target HCV *via* inhibiting the viral protease or polymerases; however, evidence has suggested that they also inhibit the function of cellular DNA polymerases, including DNA polymerase gamma (Polg), which is critical for replication of the mitochondrial genome (63–65). In this study, our HCV patients were all DAA-treatment naïve, suggesting that our observations of reduced mtDNA content in our cohort was primarily due to the HCV infection. Another biological variable that might influence the outcome of HCV disease is sex. Cross-sectional studies evaluating HCV clearance in males and females determined that females (44%) had higher rates of viral clearance than males (33.7%) (66). Females have also been shown to spontaneously clear the virus, further implicating that sex affects T cell immunity and disease outcome (67, 68). In this study, our HCV patient cohort was primarily male, thus we chose to use HS that matched our patient cohort. Additionally, HCV T cells show a reduced ability to respond to TCR stimulation. Individuals that are able to clear the virus within 6 months of infection largely rely on their T cell responses; however, if this does not occur, T cells may enter into exhaustion and senescence, leading to reduced responsiveness to TCR stimulation (39, 69). It is possible that T cell exhaustion and senescence lead to deficits in cell activation and proliferation, resulting in T cell dysfunction due to disrupted cellular ROS signaling, reduced mtDNA content, and compromised mitochondrial function.

In summary, we investigated the oxidative stress-mediated mitochondrial compromise in CD4 T cells from chronically HCV-infected individuals. We found impaired mitochondrial fitness, repression of PGC-1α and mtTFA expression, increased production of mitochondrial-specific ROS, and reduced mtDNA content. We demonstrated the role of mtTFA in mitochondrial functions and disrupted expression of mitochondrially localized proteins involved in regulating mtDNA damage repair and maintenance, responses to oxidative stress, and apoptosis. We thus conclude that chronic HCV infection alters CD4 T cell mtDNA content and metabolism *via* affecting multiple mitochondrial regulatory proteins. Therefore, strategies designed to alleviate oxidative stress and target the dysregulated mitochondrial proteins may restore T cell functions during chronic HCV infection.

## Data Availability Statement

The original contributions presented in the study are included in the article/**Supplementary Material**. Further inquiries can be directed to the corresponding author.

## Ethics Statement

The studies involving human participants were reviewed and approved by The Joint Institutional Review Board (IRB) of East Tennessee State University and James H. Quillen VA Medical Center (ETSU/VAMC). The patients/participants provided their written informed consent to participate in this study.

## Author Contributions

MS and JZ performed most of the experiments and wrote the manuscript. LW, LN, DC, XD, SK, JYZ, and YZ performed some experiments. XW provided technical support. SN, ME, and JPM offered intellectual input for troubleshooting and discussion of the findings. ZQY supervised the project and revised the manuscript with the help of all other authors. All authors contributed to the article and approved the submitted version.

## Funding 

This work was supported by National Institutes of Health grants R01AI114748, R21AI138598, R21AI157909, and R15AG069544 (to ZQY) and R15GM114716 and R15AI143377 (to JPM); VA Merit Review Awards 1I01BX002670 and 1I01BX004281 (to ZQY) and 5I01BX005428–02 (to JPM); DoD Award PR170067 (to ZQY). This publication is the result of work supported with resources and the use of facilities at the James H. Quillen Veterans Affairs Medical Center. The contents in this publication do not represent the views of the Department of Veterans Affairs or the United States Government.

## Conflict of Interest

The authors declare that the research was conducted in the absence of any commercial or financial relationships that could be construed as a potential conflict of interest.

## Publisher’s Note

All claims expressed in this article are solely those of the authors and do not necessarily represent those of their affiliated organizations, or those of the publisher, the editors and the reviewers. Any product that may be evaluated in this article, or claim that may be made by its manufacturer, is not guaranteed or endorsed by the publisher.

## References

[1] Ryerson AB Schillie S Barker LK Kupronis BA Wester C. Vital Signs: Newly Reported Acute and Chronic Hepatitis C Cases ― United States, 2009–2018. Morb Mortal Wkly Rep (2020) 69:399–404. doi: 10.15585/mmwr.mm6914a2 PMC7147907 32271725

[2] Yao ZQ Moorman JP. Immune Exhaustion and Immune Senescence: Two Distinct Pathways for HBV Vaccine Failure During HCV and/or HIV Infection. Arch Immunol Ther Exp (Warsz) (2013) 61:193–201. doi: 10.1007/s00005-013-0219-0 23400275 PMC3792483

[3] Shi L Wang JM Ren JP Cheng YQ Ying RS Wu XY. KLRG1 Impairs CD4 + T Cell Responses *via* P16 Ink4a and P27 Kip1 Pathways: Role in Hepatitis B Vaccine Failure in Individuals With Hepatitis C Virus Infection. J Immunol (2014) 192:649–57. doi: 10.4049/jimmunol.1302069 PMC3894750 24337749

[4] Moorman JP Zhang CL Ni L Ma CJ Zhang Y Wu XY. Impaired Hepatitis B Vaccine Responses During Chronic Hepatitis C Infection: Involvement of the PD-1 Pathway in Regulating CD4+ T Cell Responses. Vaccine (2011) 29:3169–76. doi: 10.1016/j.vaccine.2011.02.052 PMC3090659 21376795

[5] Wang JM Ma CJ Li GY Wu XY Thayer P Greer P. Tim-3 Alters the Balance of IL-12/IL-23 and Drives TH17 Cells: Role in Hepatitis B Vaccine Failure During Hepatitis C Infection. Vaccine (2013) 31:2238–45. doi: 10.1016/j.vaccine.2013.03.003 PMC3667544 23499521

[6] Kramer ES Hofmann C Smith PG Shiffman ML Sterling RK. Response to Hepatitis A and B Vaccine Alone or in Combination in Patients With Chronic Hepatitis C Virus and Advanced Fibrosis. Dig Dis Sci (2009) 54:2016–25. doi: 10.1007/s10620-009-0867-4 19517231

[7] Deng L Chen M Tanaka M Ku Y Itoh T Shoji I. HCV Upregulates Bim Through the ROS/JNK Signalling Pathway, Leading to Bax-Mediated Apoptosis. J Gen Virol (2015) 96:2670–83. doi: 10.1099/jgv.0.000221 26296767

[8] Piccoli C Scrima R Quarato G D’Aprile A Ripoli M Lecce L. Hepatitis C Virus Protein Expression Causes Calcium-Mediated Mitochondrial Bioenergetic Dysfunction and Nitro-Oxidative Stress. Hepatology (2007) 46:58–65. doi: 10.1002/hep.21679 17567832

[9] Gerresheim GK Roeb E Michel AM Niepmann M. Hepatitis C Virus Downregulates Core Subunits of Oxidative Phosphorylation, Reminiscent of the Warburg Effect in Cancer Cells. Cells (2019) 8:1410. doi: 10.3390/cells8111410 PMC6912740 31717433

[10] Mansouri A Gattolliat CH Asselah T. Mitochondrial Dysfunction and Signaling in Chronic Liver Diseases. Gastroenterology (2018) 155:629–47. doi: 10.1053/j.gastro.2018.06.083 30012333

[11] Brault C Lévy PL Bartosch B. Hepatitis C Virus-Induced Mitochondrial Dysfunctions. Viruses (2013) 5:954–80. doi: 10.3390/v5030954 PMC3705306 23518579

[12] Quarato G Scrima R Agriesti F Moradpour D Capitanio N Piccoli C. Targeting Mitochondria in the Infection Strategy of the Hepatitis C Virus. Int J Biochem Cell Biol (2013) 45:156–66. doi: 10.1016/j.biocel.2012.06.008 22710347

[13] Vespasiani-Gentilucci U Gallo P De Vincentis A Galati G Picardi A. Hepatitis C Virus and Metabolic Disorder Interactions Towards Liver Damage and Atherosclerosis. World J Gastroenterol (2014) 20:2825–38. doi: 10.3748/wjg.v20.i11.2825 PMC3961987 24659875

[14] Ka Yu Siu G Zhou F Kuen Yu M Zhang L Wang T Liang Y. Hepatitis C Virus NS5A Protein Cooperates With Phosphatidylinositol 4-Kinase Iiiα to Induce Mitochondrial Fragmentation. Sci Rep (2016) 6:23464. doi: 10.1038/srep23464 27010100 PMC4806301

[15] Day CL Kaufmann DE Kiepiela P Brown JA Moodley ES Reddy S. PD-1 Expression on HIV-Specific T Cells Is Associated With T-Cell Exhaustion and Disease Progression. Nature (2006) 443:350–4. doi: 10.1038/nature05115 16921384

[16] Trautmann L Janbazian L Chomont N Said EA Gimmig S Bessette B. Upregulation of PD-1 Expression on HIV-Specific CD8+ T Cells Leads to Reversible Immune Dysfunction. Nat Med (2006) 12:1198–202. doi: 10.1038/nm1482 16917489

[17] Bengsch B Seigel B Ruhl M Timm J Kuntz M Blum HE. Coexpression of PD-1, 2b4, CD160 and KLRG1 on Exhausted HCV-Specific CD8+ T Cells Is Linked to Antigen Recognition and T Cell Differentiation. PloS Pathog (2010) 6. doi: 10.1371/journal.ppat.1000947 PMC2883597 20548953

[18] Chen DY Wolski D Aneja J Matsubara L Robilotti B Hauck G. Hepatitis C Virus-Specific CD4+ T Cell Phenotype and Function in Different Infection Outcomes. J Clin Invest (2020) 130:768–73. doi: 10.1172/JCI126277 PMC6994113 31904582

[19] Nguyen LN Zhao J Cao D Dang X Wang L Lian J. Inhibition of TRF2 Accelerates Telomere Attrition and DNA Damage in Naïve CD4 T Cells During HCV Infection. Cell Death Dis (2018) 9:900. doi: 10.1038/s41419-018-0897-y 30185784 PMC6125360

[20] Zhao J Dang X Zhang P Nguyen LN Cao D Wang L. Insufficiency of DNA Repair Enzyme ATM Promotes Naive CD4 T-Cell Loss in Chronic Hepatitis C Virus Infection. Cell Discovery (2018) 4. doi: 10.1038/s41421-018-0015-4 PMC5891503 29644094

[21] Cao D Khanal S Wang L Li Z Zhao J Nguyen LN. A Matter of Life or Death: Productively Infected and Bystander CD4 T Cells in Early HIV Infection. Front Immunol (2021) 11:626431. doi: 10.3389/fimmu.2020.626431 33643305 PMC7907524

[22] Ji Y Dang X Nguyen LN Nguyen LNT Zhao J Cao D. Topological DNA Damage, Telomere Attrition and T Cell Senescence During Chronic Viral Infections. Immun Ageing (2019) 16. doi: 10.1186/s12979-019-0153-z PMC6591813 31285747

[23] Dang X Ogbu SC Zhao J Nguyen LNT Cao D Nguyen N. Inhibition of Topoisomerase IIA (Top2α) Induces Telomeric DNA Damage and T Cell Dysfunction During Chronic Viral Infection. Cell Death Dis (2020) 11:196. doi: 10.1038/s41419-020-2395-2 32193368 PMC7081277

[24] Schank M Zhao J Wang L Li Z Cao D Nguyen LN. Telomeric Injury by KML001 in Human T Cells Induces Mitochondrial Dysfunction Through the P53-PGC-1 α Pathway. Cell Death Dis (2020) 11. doi: 10.1038/s41419-020-03238-7 PMC7710715 33268822

[25] Wang L Lu Z Zhao J Schank M Cao D Dang X. Selective Oxidative Stress Induces Dual Damage to Telomeres and Mitochondria in Human T Cells. Aging Cell (2021). doi: 10.1111/ACEL.13513 PMC8672791 34752684

[26] Weinberg SE Sena LA Chandel NS. Mitochondria in the Regulation of Innate and Adaptive Immunity. Immunity (2015) 42:406–17. doi: 10.1016/j.immuni.2015.02.002 PMC4365295 25786173

[27] Sahin E Colla S Liesa M Moslehi J Müller FL Cooper M. Telomere Dysfunction Induces Metabolic and Mitochondrial Compromise. Nature (2011) 470:359–65. doi: 10.1038/nature09787.Telomere PMC3741661 21307849

[28] Correia-Melo C Passos JF. Mitochondria: Are They Causal Players in Cellular Senescence? Biochim Biophys Acta - Bioenerg (2015) 1847:1373–9. doi: 10.1016/j.bbabio.2015.05.017 26028303

[29] Zhao J Schank MB Wang L Li Z Nguyen LN Dang X. Mitochondrial Functions Are Compromised in CD4 T Cells From ART-Controlled PLHIV. Front Immunol (2021) 12:658420. doi: 10.3389/FIMMU.2021.658420 34017335 PMC8129510

[30] Rooney JP Ryde IT Sanders LH Howlett EV Colton MD Germ KE. PCR Based Determination of Mitochondrial DNA Copy Number in Multiple Species. Methods Mol Biol (2015) 1241:23–8. doi: 10.1007/978-1-4939-1875-1_3 PMC4312664 25308485

[31] Hultquist JF Hiatt J Schumann K McGregor MJ Roth TL Haas P. CRISPR–Cas9 Genome Engineering of Primary CD4 + T Cells for the Interrogation of HIV–host Factor Interactions. Nat Protoc (2019) 14:1–27. doi: 10.1038/s41596-018-0069-7 30559373 PMC6637941

[32] Levitan O Chen M Kuang X Cheong KY Jiang J Banal M. Structural and Functional Analyses of Photosystem II in the Marine Diatom Phaeodactylum Tricornutum. Proc Natl Acad Sci USA (2019) 116:17316–22. doi: 10.1073/pnas.1906726116 PMC6717305 31409711

[33] Wang T Weinman SA. Interactions Between Hepatitis C Virus and Mitochondria: Impact on Pathogenesis and Innate Immunity. Curr Pathobiol Rep (2013) 1:179–87. doi: 10.1007/s40139-013-0024-9 PMC3743448 23956955

[34] Medvedev R Ploen D Hildt E. HCV and Oxidative Stress: Implications for HCV Life Cycle and HCV-Associated Pathogenesis. Oxid Med Cell Longev (2016) 2016. doi: 10.1155/2016/9012580 PMC4756209 26955431

[35] Younes SA Talla A Ribeiro SP Saidakova EV Korolevskaya LB Shmagel KV. Cycling CD4+ T Cells in HIV-Infected Immune Nonresponders Have Mitochondrial Dysfunction. J Clin Invest (2018) 128:5083–94. doi: 10.1172/JCI120245 PMC6205369 30320604

[36] Tomkova S Misuth M Lenkavska L Miskovsky P Huntosova V. *In Vitro* Identification of Mitochondrial Oxidative Stress Production by Time-Resolved Fluorescence Imaging of Glioma Cells. Biosci Rep (2018) 1865:616–28. doi: 10.1016/j.bbamcr.2018.01.012 29410069

[37] Kweon SM Kim HJ Lee ZW Kim SJ Kim SIL Paik SG. Real-Time Measurement of Intracellular Reactive Oxygen Species Using MitoTracker Orange (CMH2TMRos). Biosci Rep (2001) 21:341–52. doi: 10.1023/A:1013290316939 11893000

[38] Kholmukhamedov A Schwartz JM Lemasters JJ. Isolated Mitochondria Infusion Mitigates Ischemia-Reperfusion Injury of the Liver in Rats: Mitotracker Probes and Mitochondrial Membrane Potential. Shock (2013) 39:543. doi: 10.1097/01.SHK.0000430660.63077.7F PMC3759289 23680774

[39] Luxenburger H Neumann-Haefelin C Thimme R Boettler T. HCV-Specific T Cell Responses During and After Chronic HCV Infection. Viruses (2018) 10. doi: 10.3390/v10110645 PMC6265781 30453612

[40] Angajala A Lim S Phillips JB Kim JH Yates C You Z. Diverse Roles of Mitochondria in Immune Responses: Novel Insights Into Immuno-Metabolism. Front Immunol (2018) 9:1605. doi: 10.3389/fimmu.2018.01605 30050539 PMC6052888

[41] Nguyen LN Nguyen LNT Zhao J Schank M Dang X Cao D. Immune Activation Induces Telomeric DNA Damage and Promotes Short-Lived Effector T Cell Differentiation in Chronic HCV Infection. Hepatology (2021) 74:2380–94. doi: 10.1002/hep.32008 PMC8542603 34110660

[42] Khan NA Nikkanen J Yatsuga S Jackson C Wang L Pradhan S. Mtorc1 Regulates Mitochondrial Integrated Stress Response and Mitochondrial Myopathy Progression. Cell Metab (2017) 26:419–428.e5. doi: 10.1016/j.cmet.2017.07.007 28768179

[43] Lai ZW Kelly R Winans T Marchena I Shadakshari A Yu J. Sirolimus in Patients With Clinically Active Systemic Lupus Erythematosus Resistant to, or Intolerant of, Conventional Medications: A Single-Arm, Open-Label, Phase 1/2 Trial. Lancet (2018) 391:1186–96. doi: 10.1016/S0140-6736(18)30485-9 PMC5891154 29551338

[44] Paracha UZ Fatima K Alqahtani M Chaudhary A Abuzenadah A Damanhouri G. Oxidative Stress and Hepatitis C Virus. Virol J (2013) 10:251. doi: 10.1186/1743-422X-10-251 23923986 PMC3751576

[45] Devadas S Zaritskaya L Rhee SG Oberley L Williams MS. Discrete Generation of Superoxide and Hydrogen Peroxide by T Cell Receptor Stimulation: Selective Regulation of Mitogen-Activated Protein Kinase Activation and Fas Ligand Expression. J Exp Med (2002) 195:59–70. doi: 10.1084/jem.20010659 11781366 PMC2196010

[46] Hildeman DA Mitchell T Teague TK Henson P Day BJ Kappler J. Reactive Oxygen Species Regulate Activation-Induced T Cell Apoptosis. Immunity (1999) 10:735–44. doi: 10.1016/S1074-7613(00)80072-2 10403648

[47] Schank M Zhao J Moorman JP Yao ZQ. The Impact of HIV- and ART-Induced Mitochondrial Dysfunction in Cellular Senescence and Aging. Cells (2021) 10. doi: 10.3390/cells10010174 PMC7830696 33467074

[48] Ivanov AV Smirnova OA Petrushanko IY Ivanova ON Karpenko IL Alekseeva E. HCV Core Protein Uses Multiple Mechanisms to Induce Oxidative Stress in Human Hepatoma Huh7 Cells. Viruses (2015) 7:2745–70. doi: 10.3390/v7062745 PMC4488712 26035647

[49] Abdalla MY Ahmad IM Spitz DR Schmidt WN Britigan BE. Hepatitis C Virus-Core and non Structural Proteins Lead to Different Effects on Cellular Antioxidant Defenses. J Med Virol (2005) 76:489–97. doi: 10.1002/JMV.20388 15977232

[50] Gong G Waris G Tanveer R Siddiqui A. Human Hepatitis C Virus NS5A Protein Alters Intracellular Calcium Levels, Induces Oxidative Stress, and Activates STAT-3 and NF-κb. Proc Natl Acad Sci USA (2001) 98:9599. doi: 10.1073/PNAS.171311298 11481452 PMC55498

[51] Lozano-Sepulveda SA Bryan-Marrugo OL Cordova-Fletes C Gutierrez-Ruiz MC Rivas-Estilla AM. Oxidative Stress Modulation in Hepatitis C Virus Infected Cells. World J Hepatol (2015) 7:2880. doi: 10.4254/WJH.V7.I29.2880 26692473 PMC4678374

[52] Negash AA Olson RM Griffin S Gale M. Modulation of Calcium Signaling Pathway by Hepatitis C Virus Core Protein Stimulates NLRP3 Inflammasome Activation. PloS Pathog (2019) 15:e1007593. doi: 10.1371/JOURNAL.PPAT.1007593 30811485 PMC6392285

[53] Chen W Xu Y Li H Tao W Xiang Y Huang B. HCV Genomic RNA Activates the NLRP3 Inflammasome in Human Myeloid Cells. PloS One (2014) 9:e84953. doi: 10.1371/JOURNAL.PONE.0084953 24400125 PMC3882267

[54] Kannan RP Hensley LL Evers LE Lemon SM McGivern DR. Hepatitis C Virus Infection Causes Cell Cycle Arrest at the Level of Initiation of Mitosis. J Virol (2011) 85:7989–8001. doi: 10.1128/JVI.00280-11 21680513 PMC3147967

[55] Walters KA Syder AJ Lederer SL Diamond DL Paeper B Rice CM. Genomic Analysis Reveals a Potential Role for Cell Cycle Perturbation in HCV-Mediated Apoptosis of Cultured Hepatocytes. PloS Pathog (2009) 5:e1000269. doi: 10.1371/JOURNAL.PPAT.1000269 19148281 PMC2613535

[56] Barili V Fisicaro P Montanini B Acerbi G Filippi A Forleo G. Targeting P53 and Histone Methyltransferases Restores Exhausted CD8+ T Cells in HCV Infection. Nat Commun (2020) 11. doi: 10.1038/s41467-019-14137-7 PMC6992697 32001678

[57] Cao D Zhao J Nguyan LN Nguyen LNT Khanal S Dang X. Disruption of Telomere Integrity and DNA Repair Machineries by KML001 Induces T Cell Senescence, Apoptosis, and Cellular Dysfunctions. Front Immunol (2019) 10:1152. doi: 10.3389/fimmu.2019.01152 31191531 PMC6540964

[58] Qian W Kumar N Roginskaya V Fouquerel E Opresko PL Shiva S. Chemoptogenetic Damage to Mitochondria Causes Rapid Telomere Dysfunction. Proc Natl Acad Sci USA (2019) 116:18435–44. doi: 10.1073/pnas.1910574116 PMC6744920 31451640

[59] Fouquerel E Barnes RP Uttam S Watkins SC Bruchez MP Opresko PL. Targeted and Persistent 8-Oxoguanine Base Damage at Telomeres Promotes Telomere Loss and Crisis. Mol Cell (2019) 214:796–809. doi: 10.1016/j.molcel.2019.04.024 PMC6625854 31101499

[60] Hoare M Gelson WTH Das A Fletcher JM Davies SE Curran MD. CD4+ T-Lymphocyte Telomere Length Is Related to Fibrosis Stage, Clinical Outcome and Treatment Response in Chronic Hepatitis C Virus Infection. J Hepatol (2010) 53:252–60. doi: 10.1016/j.jhep.2010.03.005 PMC2913243 20462651

[61] El-Hattab AW Craigen WJ Scaglia F. Mitochondrial DNA Maintenance Defects. Biochim Biophys Acta - Mol Basis Dis (2017) 1863:1539–55. doi: 10.1016/j.bbadis.2017.02.017 28215579

[62] Stewart JB Chinnery PF. The Dynamics of Mitochondrial DNA Heteroplasmy: Implications for Human Health and Disease. Nat Rev Genet (2015) 16:530–42. doi: 10.1038/nrg3966 26281784

[63] Pinti M Salomoni P Cossarizza A. Anti-HIV Drugs and the Mitochondria. Biochim Biophys Acta - Bioenerg (2006) 1757:700–7. doi: 10.1016/j.bbabio.2006.05.001 16782042

[64] Durand M Nagot N Nhu QBT Vallo R Thuy LLT Duong HT. Mitochondrial Genotoxicity of Hepatitis C Treatment Among People Who Inject Drugs. J Clin Med (2021) 10:4824. doi: 10.3390/JCM10214824 34768343 PMC8584601

[65] Aregay A Owusu Sekyere S Deterding K Port K Dietz J Berkowski C. Elimination of Hepatitis C Virus has Limited Impact on the Functional and Mitochondrial Impairment of HCV-Specific CD8+ T Cell Responses. J Hepatol (2019) 71:889–99. doi: 10.1016/j.jhep.2019.06.025 31295532

[66] Stroffolini T Rapicetta M Di Stefano R. Hepatitis C Virus Clearance and Gender. Gut (2007) 56:884. doi: 10.1136/gut.2006.116632 PMC1954870 17519492

[67] Baden R Rockstroh JK Buti M. Natural History and Management of Hepatitis C: Does Sex Play a Role? J Infect Dis (2014) 209:S81–5. doi: 10.1093/infdis/jiu057 24966194

[68] Grebely J Page K Sacks-Davis R van der Loeff MS Rice TM Bruneau J. The Effects of Female Sex, Viral Genotype, and IL28B Genotype on Spontaneous Clearance of Acute Hepatitis C Virus Infection. Hepatology (2014) 59:109–20. doi: 10.1002/HEP.26639 PMC3972017 23908124

[69] Thimme R. T Cell Immunity to Hepatitis C Virus: Lessons for a Prophylactic Vaccine. J Hepatol (2021) 74:220–9. doi: 10.1016/j.jhep.2020.09.022 33002569

