# Supplementary material for: Oxidative Stress Induces Mitochondrial Compromise in CD4 T Cells From Chronically HCV-Infected Individuals
Source: Front Immunol. 2021 Dec 8;12:760707. doi: 10.3389/fimmu.2021.760707 (PMC8692574; doi:10.3389/fimmu.2021.760707)
Supplement: Supplementary file 1 [file DataSheet_1.docx]

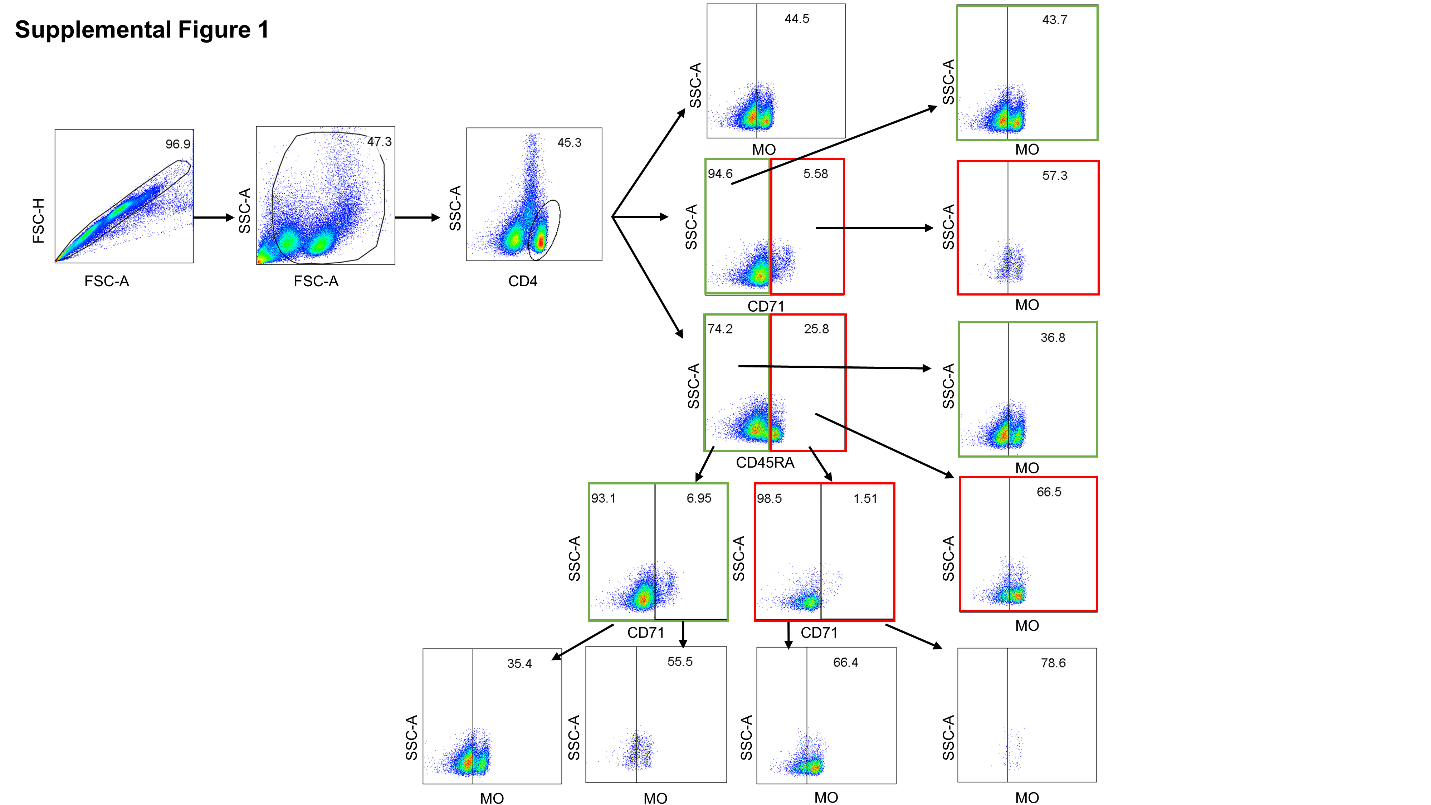


**Supplemental Fig.1.** Flow cytometry gating strategy. Representative pseudocolor plots for flow cytometry grating strategy are shown. Briefly, CD4^+^ cells were gated after gating singlets and scatter in PBMCs from HS or chronically HCV-infected individuals. Next, the expression of MO, CD71, and CD45RA was analyzed in CD4^+^ cells. The expression of CD71 was further analyzed in CD4^+^ CD45RA^+^ and CD4^+^ CD45RA^-^ cell subsets. Lastly, MO expression was determined in CD4^+^ CD71^-^, CD4^+^ CD71^+^, CD4^+^ CD45RA^-^, CD4^+^ CD45RA^+^, CD4^+^ CD45RA^-^ CD71^-^, CD4^+^ CD45RA^-^ CD71^+^, CD4^+^ CD45RA^+^ CD71^-^, and CD4^+^ CD45RA^+^ CD71^+^ cell subsets. Similar to MO staining, PBMCs were stained with MG, anti-PGC1α, anti-mtTFA, along with anti-CD4, anti-CD45RA, and anti-CD71 antibodies.


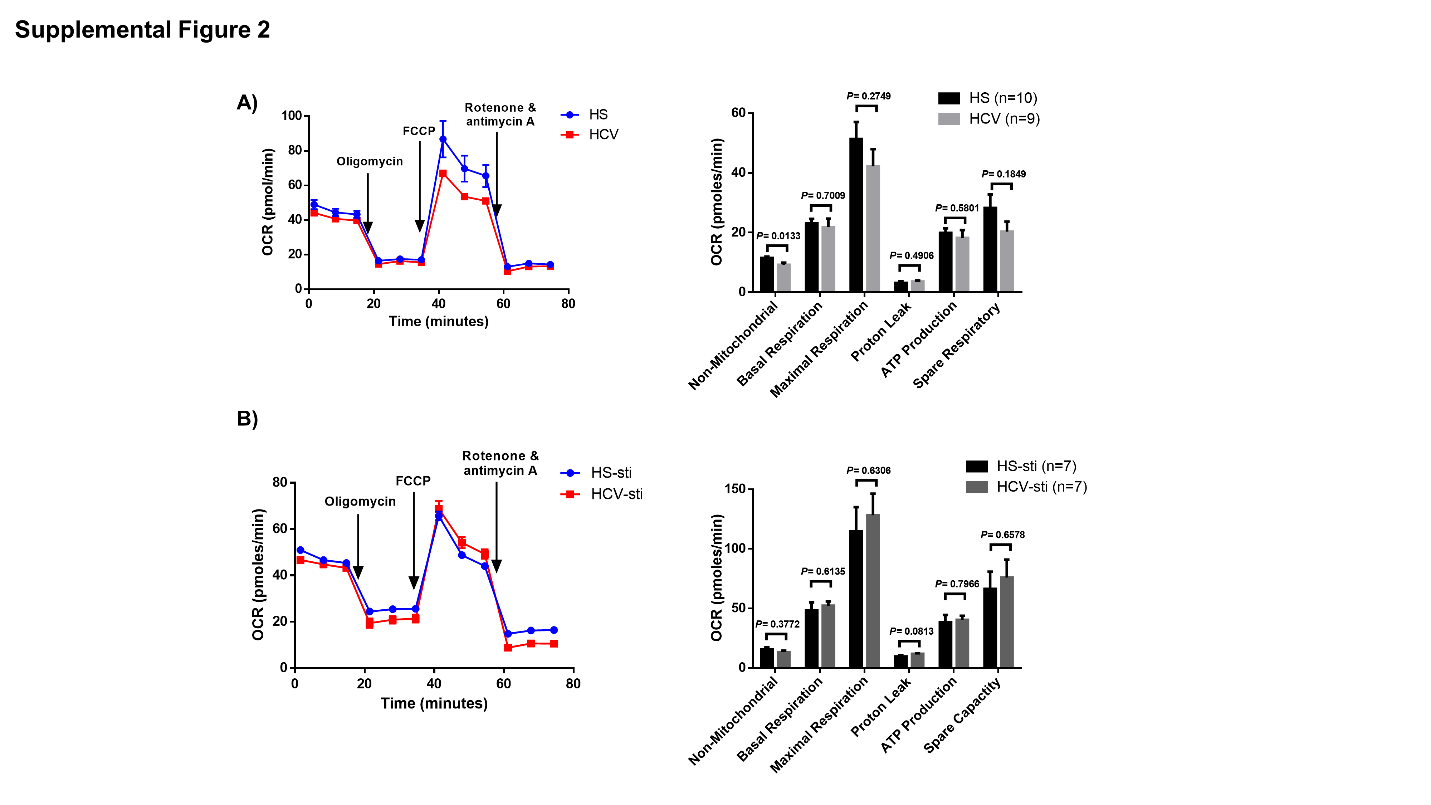


**Supplemental Fig.2. Cellular respiration in CD4 T cells from HCV-infected individuals and HS. A-B)** Representative and summary OCR for non-mitochondrial, basal respiration, maximal respiration, spare capacity, proton leak, and ATP production in unstimulated (*A*) or TCR-stimulated CD4 T cells (*B*) from HCV-infected individuals and HS. The data were analyzed by parametric unpaired T-tests.
